# P-24. Toward the clinical development of ATI-1701, a genetically defined live attenuated tularemia vaccine

**DOI:** 10.1093/ofid/ofae631.231

**Published:** 2025-01-29

**Authors:** Gary S Nabors, Arthur Baran, Carl Gelhaus

**Affiliations:** Appili Therapeutics, Laytonsville, Maryland; Appili Therapeutics, Laytonsville, Maryland; Appili Therapeutics, Laytonsville, Maryland

## Abstract

**Background:**

Tularemia is a life-threatening disease caused by the bacterium *Francisella tularensis*. While rare, the disease is notable for its wide host range, low infectious dose, high case fatality rate, and having been previously weaponized. Type A (*F. t. tularensis*) strains are found predominantly in North America and are generally more virulent than Type B (*F. t. holarctica*) strains. Both a killed whole cell (KWC) and a live attenuated (LA) vaccine were initially developed and underwent clinical testing in the pre-antibiotic era. While both types of vaccines generated strong antibody responses, later human challenge studies demonstrated that the KWC vaccine failed to protect against aerosol exposure. The LA strain designated LVS, which provided better protection against aerosol challenge but less robust protection against type A strains, was used until recently to immunize laboratory workers. Obstacles for approval of LVS include concerns regarding its undefined attenuation and potential for reversion to virulence. No acellular vaccines have proven to be as effective as LA vaccines.

**Methods:**

ATI-1701 is a next-generation LA vaccine with a targeted, well-characterized mechanism of attenuation. ATI-1701 carries a *clpB* gene deletion, which is thought to inhibit its ability to escape from phagosomes, thereby limiting replication in host macrophages.

**Results:**

Mice immunized ID with 10 CFU of ATI-1701 were fully protected from lethal challenge with the SCHU S4 strain. Rats vaccinated with one dose of ATI-1701 and challenged via aerosol exposure to 5,600 LD50 of virulent SCHU S4 survived infection up to one year post-challenge, whereas all unvaccinated rats died. Regardless of immunizing dose (10^3^-10^9^ CFU), spleens from vaccinated rats contained 10^3^-10^5^ CFU of ATI-1701 14 days after vaccination, and bacteria were cleared from the spleen prior to day 42. Immunization of cynomolgus macaques with a single dose of 10^5^ CFU ATI-1701 protected 80-100% of animals challenged with up to 157,000 LD50 of SCHU S4 via aerosol exposure up to three months after vaccination. One year after vaccination approximately 30% of vaccinated macaques survived challenge whereas control animals died.

**Conclusion:**

ATI-1701 is a promising tularemia vaccine candidate and is being positioned for a Phase 1 clinical trial.

**Disclosures:**

**All Authors**: No reported disclosures

